# Determination and Comparison of the Pathogen Spectrum Evaluated by Microbial Culture and Multiplex PCR During Bronchoscopy with Regard to Clinical Utility of Routine Bronchial Wash in Patients with Various Pulmonary Diseases

**DOI:** 10.3390/diagnostics15040469

**Published:** 2025-02-14

**Authors:** Christopher Milacek, Christina Bal, Peter Starzengruber, Sonja Zehetmayer, Marco Idzko, Daniela Gompelmann

**Affiliations:** 1 Division of Pulmonology, Department of Internal Medicine II, Medical University of Vienna, 1090 Vienna, Austria; christopher.milacek@meduniwien.ac.at (C.M.); marco.idzko@meduniwien.ac.at (M.I.); 2 Division for Clinical Microbiology, Department of Laboratory Medicine, Medical University of Vienna, 1090 Vienna, Austria; peter.starzengruber@meduniwien.ac.at; 3 Center for Medical Data Science, Institute of Medical Statistics, Medical University of Vienna, 1090 Vienna, Austria; sonja.zehetmayer@meduniwien.ac.at

**Keywords:** bronchial wash, clinical utility, PCR, microbial culture, pathogen spectrum, pulmonary infection

## Abstract

**Background/Objectives:** Bronchial wash for microbiological cultivation and/or multiplex polymerase chain reaction (PCR) is often routinely performed during bronchoscopy independent of the indication for bronchoscopy. This study aimed to determine and compare the pathogen spectrum evaluated by culture and PCR in bronchial secretion samples with regard to the underlying lung pathology. **Methods:** In this retrospective analysis, microbiological results from bronchial washes performed from February 2022 to September 2022 were collected in 245 patients (48.2% male, mean age 63.0 ± 13.9 years). Samples were assessed for bacteria/fungi culture and reported depending on the underlying pathology. Additional PCR was performed in 216 patients and compared to the results of the culture. **Results:** Cultivation and PCR revealed positive results in 20% and 24.5% of the cases, respectively. Microbiological culture most likely revealed a positive result in patients with hemoptysis (44.4%) and pulmonary infections (29.6%) among various indications for bronchoscopy, and PCR most likely identified a pathogen in patients with hemoptysis (66.7%) and COPD (36.4%). Active smoking and an increased CRP were revealed as significant predictors for a positive culture (*p* < 0.0001 and *p* = 0.021). The concordance rate between culture and PCR for identifying pathogenic microorganisms was 84.3%, resulting in a moderate agreement (kappa coefficient 0.54 [95% CI: 0.34–0.68]). **Conclusion:** PCR and culture moderately agreed, showing additional PCR testing only to be beneficial if restricted to proper indications, providing rapid diagnosis and therefore leading to immediate therapeutic decisions.

## 1. Introduction

Bronchoscopy is one of the most important methods in the diagnosis of lung diseases. Common indications are histological confirmation of suspicious pulmonary masses, mediastinal or hilar lymphadenopathy, or interstitial lung diseases. In addition, bronchoscopy offers a wide range of therapeutic options such as the implantation of valves in patients with advanced emphysema, or thermoplasty in patients with bronchial asthma.

Furthermore, bronchoscopy provides a useful tool for collecting bronchial secretions for microbiological assessment. This is particularly required in patients suffering from pneumonia with no response to antibiotic treatment, and those who are prone to colonisation and/or infection with particular pathogens e.g., immunocompromised patients [1,2]. So far, the identification of pneumonia-causing bacteria is mainly based on microbiological cultures from bronchial washes that offer results after 24–48 h. Multiplex polymerase chain reaction (PCR) is used as a complementary method to microbiological culture. Multiplex PCR provides a faster, more sensitive and specific method for identifying pathogens in lower respiratory tract infections and pneumonia. Results are available within hours compared to the 24–48 h required for culture results. Several studies have demonstrated that the use of multiplex PCR assays reduces antibiotic initiation and decreases the duration of antibiotic therapy [3,4,5,6,7].

So far, the use of multiplex PCR assays for bronchial secretion has been only reported in patients with lower respiratory tract infections or pneumonia [3,4,5]. However, a bronchial wash for microbiological cultivation is often performed during each diagnostic or therapeutic bronchoscopy in patients, regardless of the clinical suspicion of an acute respiratory infection. This retrospective study aimed to determine and compare the pathogen spectrum evaluated by microbial culture and multiplex PCR during bronchoscopy with regard to the clinical utility of routine secretion sampling in patients with various pulmonary pathologies.

## 2. Materials and Methods

The primary endpoint of this retrospective study was to evaluate the spectrum of pathogens in the bronchial secretions assessed by microbiological cultivation and by multiplex PCR in patients with different lung pathologies. All patients (n = 245) who underwent bronchoscopy at the Clinical Division of Pulmonology, Department of Internal Medicine II, at the Medical University of Vienna from February 2022 until September 2022, were enrolled in this retrospective trial. There were no exclusion criteria. The ethics committee of the Medical University of Vienna approved the protocol of this retrospective study (1751/2022). Written informed consent was not required due to the retrospective study design.

### 2.1. Data Collection and Sampling Procedure

Medical records, radiological examinations, laboratory tests, and microbiological and multiplex PCR analyses were obtained from 245 patients who underwent bronchoscopy for evaluation of different lung pathologies.

The bronchoscopy was performed either under general anesthesia or deep sedation. Most of the patients received additional local anesthesia using a topical xylocaine spray. After visual assessment of the central airways, a bronchial wash was performed by instillation of 20–40 mL of 0.9% saline solution, followed by a direct aspiration from the large airways of the respiratory tract through the working channel of the bronchoscope. Briefly, all of the samples were analysed as part of routine bacterial and fungal identification, and antibiotic resistance testing, according to the manufacturer’s instructions and microbiological quality standard (Clinical Microbiology Procedures Handbook, 4th Edition, 2016 and European Committee on Antimicrobial Susceptibility Testing, EUCAST).

When growth was observed on the culture plates after incubation, identification was performed by matrix-assisted laser desorption ionization time-of-flight mass spectrometry (MALDI-TOF MS, MALDI Biotyper, Bruker, Germany).

Microbiological cultures were considered negative when they showed no growth other than the expected resident oropharyngeal flora. In 216 out of the 245 patients, an additional multiplex polymerase chain reaction (PCR) assay was performed according to manufacturer’s instructions (BioFire^®^ FilmArray^®^ 2.0 Operator’s Manual IVD). The multiplex PCR assay (BioFire^®^ FilmArray^®^ Pneumonia Panel plus) detects 19 different bacteria and 8 viruses that primarily cause pneumonia. Within our patient cohort that was evaluated during the course of this study, the assay tested positive for the following pathogens: *Staphylococcus aureus*, *Streptococcus pneumoniae*, *Streptococcus agalactiae*, *Haemophilus influenzae*, *Proteus mirabilis*, *Pseudomonas aeruginosa*, *Moraxella catarrhalis*, *Klebsiella pneumoniae*, *Escherichia coli*, *Serratia marcescens*, coronavirus (including the following serological variants: 229E, HKU1, OC43, NL63), adenovirus, rhinovirus/enterovirus, and parainfluenza virus.

### 2.2. Statistical Analysis

Mean and standard deviation (or median and interquartile range (IQR) for skewed variables) were calculated for continuous variables for each category of pathogen, and *t*-tests (or Mann-Whitney U tests) were performed to investigate whether significant differences were present. For categorical variables, frequencies and percentages of pathogens and corresponding chi-square tests were calculated (or Fisher exact tests in the case of limited observations). The same computations were then repeated for the dichotomous variable Biofire.

A multiple logistic regression model was performed including all the variables with a *p*-value < 0.05 from the univariate analyses. The *p*-values, odds ratios (OR), and the corresponding 95% confidence interval (CI) were calculated.

A contingency table for the two variables “positive culture” and “positive multiplex PCR” was generated, and the kappa coefficient as a measure of agreement and the corresponding 95% confidence interval (CI) were calculated.

Due to the exploratory and retrospective character of this study, statistical significance was considered for *p*-values smaller than 0.05, but not in a confirmatory manner. Thus, no adjustment for multiplicity was performed.

All analyses were performed using the R program (R 4.2.2).

## 3. Results

### 3.1. Patient Characteristics and Indication for Bronchoscopy

In this retrospective study, 245 patients (48.2% male, mean age 63.0 ± 13.9 years) who underwent bronchoscopy at the Clinical Division of Pulmonology, Department of Internal Medicine II, Medical University of Vienna, from February 2022 until September 2022 were enrolled.

With a rate of 46.2%, histological sampling of pulmonary lesions suspicious for malignant disease, re-biopsy, or mediastinal staging in proven malignancies represented the most common indication for bronchoscopy. The second most frequent indication for bronchoscopy was the performance of bronchoalveolar lavage and/or transbronchial cryobiopsy in interstitial lung disease. Clinical characteristics and indication for bronchoscopy are presented in Table 1.

### 3.2. Results of Microbiological Culture and Multiplex PCR Assessed with Regard to Indication for Bronchoscopy

The microbiological culture and multiplex PCR assays revealed pathogenic microorganisms in 20% (49/245) and in 24.5% (53/216) of the cases, respectively. Considering the indication for the bronchoscopy and thus the predominant underlying lung disease, the microbiological cultures and multiplex PCRs revealed positive results for 44.4% (4/9) and 66.7% (4/6) of patients with hemoptysis, and for 29.6% (8/27) and 33.3% (9/27) of patients with pulmonary infections, respectively. For patients with pulmonary masses suspicious for malignancy, the cultures and PCRs revealed pathogenic microorganisms in 18.9% (24/127 and 21/111) of the subjects. Pathogen-positive results for the cultures and PCRs were found in 14.3% (2/14) and 36.4% (4/11) of patients with COPD, in 21.4% (3/14) and 30.8% (4/13) of patients with asthma, in 12.3% (8/65) and 24.1% (14/58) of patients with ILD, and in 20% (2/10) and 22.2% (2/9) of patients with long COVID-19, respectively. No pathogens were detected in 1 patient with a chronic cough. In the patients with hemoptysis, gram-negative bacteria were the predominant isolated pathogens. Patients with malignant diseases presented the highest diversity. Table 2a,b presents the results of the microbiological cultures and multiplex PCR assays.

### 3.3. Comparison of Microbiological Culture and Multiplex PCR

An additional multiplex PCR was performed in 216 patients. Out of these patients, concordance in identifying pathogenic microorganisms was shown in 182 cases, resulting in a concordance rate of 84.3%. Thus, the overall agreement was found to be moderate with a Cohen’s kappa of 0.54 (95% CI: 0.34–0.68) (Table 3a,b).

In 34 patients, discordant results were found between the microbiological culture and multiplex PCR results. Positive PCR results but negative cultures were observed in 21 patients (Table 3a). In 4 out of these 21 patients, viruses (coronavirus, parainfluenza virus, and rhino/enterovirus) were the only pathogen detected via PCR. Since no viral cultures were performed, this contributed to the disagreement between the Biofire^®^ and the culture results. Bacteria found in the remaining 17 patients are listed in Supplemental Table S1.

In 13 cases, bacteria and fungi were detected by traditional culture but not by PCR, whereas in 5 out of the 13 cases, the following pathogens were not tested by PCR: *Enterococcus faecium*, *Haemophilus parainfluenza*, *Citrobacter koseri*, *Aspergillus fumigatus*, and mycobacteria Table 3a). Bacteria found in the remaining 8 cases are listed in the Supplemental Table S2.

After excluding all of the patients with viruses as causal pathogens, or with bacteria detected by culture that were not listed in the PCR panel, the concordance rate between the traditional culture and multiplex PCR was 87.7% (Table 3b). Identical microorganisms were found in 96% (27/28), whereas in 3 cases the PCR revealed more than one microorganism that were not detected by culture. A positive culture and positive PCR revealed different bacteria in only one patient. The comparison of BioFire^®^ FilmArray^®^ Pneumonia Panel to conventional culture for each microorganism is presented in Table 4.

### 3.4. Predictors for Positive Culture or Positive Multiplex PCR Assay

Comparing patients with a positive culture to those with no detected pathogenic microorganisms, smoking status and CRP levels differed significantly. The cultures revealed positive results significantly more often in patients with an active smoking status compared to former smokers or non-smokers (*p* < 0.001), and in patients with an increased CRP (*p* = 0.036). A multiple logistic regression analysis confirmed their predictive character (*p* = 0.0001 and *p* = 0.0210) (Table 5). For the multiplex PCR assay, only hemoptysis as the indication for bronchoscopy was found to be a predictor (*p* = 0.033). Because the patient cohort was very small, this result must be interpreted with caution.

## 4. Discussion

Bronchoscopy is one of the most important diagnostic tools for identifying various pulmonary diseases. Pulmonary nodules suspicious for malignant diseases, interstitial lung disease, and pulmonary infiltrations due to infectious or non-infectious origin are the most common indications for a diagnostic bronchoscopy. Moreover, bronchoscopy also offers a therapeutic option, e.g., for patients with advanced emphysema, asthma, or chronic bronchitis. During bronchoscopic procedures, an additional bronchial wash for microbiological assessment is often routinely performed, often without regard to the indication for bronchoscopy.

In this retrospective study, we examined the impact of a bronchial wash for microbiological assessment in all patients undergoing bronchoscopy. Further, we compared the results of traditional cultures to BioFire^®^ FilmArray^®^ Pneumonia Panels. To our knowledge, this is the first trial to evaluate the impact of a multiplex PCR assay in bronchoscopically obtained secretion samples from patients without pneumonia.

Standard culture and multiplex PCR assays revealed positive results in 20.2% and 24.5%, respectively. It is reasonable that the greatest microorganism detection rate was found in patients with pulmonary infections, but also in patients with hemoptysis because these conditions might additionally serve as a media for bacterial growth. An active smoker status and an increased CRP were identified as predictors for a positive traditional culture.

This finding clearly demonstrates that the indication for a bronchial wash should be restricted to situations in which an infectious disease is proven, suspected, or expected. In addition, it should always be considered whether the detection of a microorganism influences the clinical outcome. In patients with pneumonia, the value of bronchoscopy is well known. In a prospective study of 262 patients with community-acquired pneumonia, van der Eerden and coauthors described that bronchoscopy was of additive diagnostic value in 49% of the patients who did not expectorate sputum, and in 52% of those in whom treatment had failed [8]. In other pulmonary diseases, it is also known that infection with some pathogens (e.g., Pseudomonas aeruginosa) can cause worse lung inflammation and symptoms, with a deterioration in clinical status [9]. Therefore, when performing a bronchial wash, it should always be considered: (1) how high is the probability of detecting a microorganism, and (2) would evidence of a microorganism have an impact on the clinical outcome?

Moreover, our data revealed that a different pathogen spectrum can be expected in different lung pathologies. Therefore, it could be considered whether microbiological testing should be limited to a specific pathogen spectrum in patients with underlying lung diseases.

With this study, we compared the detection rate of pathogens using multiplex PCR assays against conventional culture and found an overall positive agreement of 84% for detecting pathogenic microorganisms. After excluding all patients with viruses as causal pathogens, or with bacteria detected by cultures not listed in the PCR panel, the concordance rate between traditional cultures and multiplex PCR increased to 88%. These findings are similar to other studies [5,6]. Li-Geng et al. reported an agreement of 77% when comparing traditional bacterial and viral culture methods with multiplex PCR assays in the bronchoalveolar lavage of 24 immunocompromised patients with suspected pneumonia [5]. In a prospective study, Lee and colleagues revealed an overall positive agreement of 79% between BioFire^®^ FilmArray^®^ Pneumonia panels and traditional culture in 51 patients with respiratory tract infections [6].

Comparable to other studies, our analysis revealed a higher sensitivity for multiplex PCR to detect microorganisms (20.2% vs. 24.5%), corroborating the results of Baudel and colleagues who compared culture and multiplex PCR in 65 critically ill patients with pneumonia [7]. The pathogen identification rate with PCR was greater than with culture (66% vs. 40%). Tschiedel and colleagues described the superiority of PCR in comparison to culture in a retrospective analysis including 70 children with pulmonary infections [10]. Pathogens were detected more frequently by PCR than by culture (83% vs. 31%). Mitton et al. compared the BioFire^®^ FilmArray^®^ Pneumonia plus array with conventional culture in 59 patients with lower respiratory tract infections and described a lower microorganism detection rate by culture [11].

These findings correspond to an improvement of pathogen detection in bloodstream infections by PCR in patients with nosocomial infections [12], sepsis [13,14] and hematological diseases, and immunocompromised patients [15].

In this trial, multiplex PCR revealed positive results in 17 patients where conventional culture showed no bacterial growth. One could assume false positives, arguing that the detection of DNA does not necessarily represent ongoing infection, as opposed to colonisation, or the remains of a previous infection. On the other hand, prior exposure to antimicrobial therapy is the most common cause of negative cultures and an absent bacterial growth. However, this assumption may be inappropriate because the positive PCR assay was compared to a less sensitive test.

Up until now, the analysis of bacterial load via traditional culture has always been considered superior to PCR because quantitative measurement allows comparison with the resident flora, thus facilitate interpretation. Aiming to overcome the disadvantage of missing the analysis of pathogen quantity by PCR, molecular methods have been introduced that provide a semi-quantitative analysis of bacterial amounts by counting bacterial nucleic acid copy numbers. Yoo and colleagues demonstrated in a retrospective trial that a semi-quantitative analysis of bacterial nucleic acid amounts by Biofire^®^ FilmArray^®^ PCR revealed that 88% of the identified bacteria with ≥10^6^ copies/mL also gave culture-positive results with significant amounts of bacteria [16]. This finding may help to determine whether the organism identified is a coloniser, or an actual pathogen.

Furthermore, traditional culture holds another advantage with the assessment of antibiotic resistance. Although PCR assays also detect resistance genes, it is recommended to use PCR only as an adjunct to microbial culture to determine susceptibility or resistance, due to the inability of PCR assays to link resistance genes to a single pathogen [16].

Finally, when comparing traditional culture and multiplex PCR, it must be noted that culture was able to detect organisms that were not included in the PCR panel. In our analysis, culture revealed an organism that was not tested by PCR in 5 cases.

The limitations of this study is its retrospective design and the small sample size. However, to our knowledge, this is the first trial that has evaluated the pathogen detection rate by culture and multiplex PCR in different lung diseases. Moreover, we used a multiplex PCR panel for pneumonia, not a respiratory panel, so that some pathogens were not evaluated.

## 5. Conclusions

In summary, bronchial washes should be restricted to situations in which an infectious microorganism is suspected to be the causative agent of the lung disease. In addition to the indication for bronchoscopy, smoking status and C-reactive protein levels should be taken into consideration. With the right indication, traditional culture is still the gold standard for culture-detectable pathogens. An additional multiplex PCR assay that provides early information on pathogens should be considered in cases where a rapid diagnosis/treatment is needed, or when pathogens not detectable by conventional culture are suspected. Multiplex PCR does not replace conventional microbial culture for detecting pathogenic microorganisms because it may not list some important, clinically relevant pathogens, or draw attention to irrelevant pathogens, as mentioned above, for example in the case of colonisation. Furthermore, PCR does not replace antimicrobial susceptibility testing by culture because only the most important resistance markers can be determined by PCR.

## Figures and Tables

**Table 1:** Clinical characteristics of 245 patients.

Patient’s Characteristics	
Sex (male, %)	48.2
Age (years; mean ± STD)	63.0 ± 13.9
**Smoking status (n; %)**	
Non-smoker	70 (28.6)
Ex-smoker	102 (41.6)
Smoker	73 (29.8)
**Indication for bronchoscopy (n; %)**	
COPD	14 (5.1)
ILD	64 (23.3)
Pulmonary lesion suspicious for malignant disease	127 (46.2)
Asthma	14 (5.1)
Chronic cough	1 (0.36)
Pulmonary infection	27 (9.8)
Hemoptysis	9 (3.2)
Long COVID-19	10 (3.6)
Other	9 (3.3)
**Laboratory tests (mean ± STD)**	
Leucocyte count	7.8 ± 2.8
CRP (mg/dL)	1.8 ± 3.7
Fibrinogen (mg/dL)	426.2 ± 117.1

**Table 2:** (a) Culture results depending on underlying predominant lung disease; (b) Multiplex PCR assay results depending on underlying predominant lung disease.

(a)								
	**COPD** **n = 14**	**Asthma** **n = 14**	**ILD** **n = 65**	**Malignant Disease** **n = 127**	**Pulmonary Infection** **n = 27**	**Chronic Cough** **n = 1**	**Hemoptysis** **n = 9**	**Long COVID-19** **n = 10**
**Positive culture, n (%)**	2 (14.3)	3 (21.4)	8 (12.3)	24 (18.9)	8 (29.6)	0 (0)	4 (44.4)	2 (20)
**Gram-positive bacteria, n**								
*Staphylococcus aureus*		1	3	1	2			1
*Streptococcus pneumoniae*		1	1	4				
*Streptococcus agalactiae*								
*Corynebacterium*				2				
*Enterococcus faecium*				1				
**Gram-negative bacteria, n**								
*Haemophilus influenza*			2	9			2	1
*Haemophilus parainfluenza*				1				
*Proteus mirabilis*				1			1	
*Pseudomonas aeruginosa*	2			2	3			
*Moraxella catarrhalis*				2			1	
*Klebsiella pneumoniae*				1				
*Klebsiella oxytoca*				1				
*Escherichia coli*		1		3	1			
*Serratia marcescens*				1				
*Citrobacter coseri*			1	1				
*Enterobacter cloacae*			1					
**Mycobacteria, n**								
*Mycobacterium tuberculosis*					1			
*Nontuberculous mycobacteria*							1	
**Fungi, n**								
*Aspergillus fumigatus*	1			1	2			
(b)								
	**COPD** **n = 11**	**Asthma** **n = 13**	**ILD** **n = 58**	**Malignant disease** **n = 111**	**Pulmonary infection** **n = 27**	**Chronic cough** **n = 1**	**Hemoptysis** **n = 6**	**Long COVID-19** **n = 9**
**Positive PCR assay, n (%)**	4 (36.4)	4 (30.8)	14 (24.1)	21 (18.9)	9 (33.3)	0 (0)	4 (66.7)	2 (22.2)
**Gram-positive bacteria, n**								
*Staphylococcus aureus*	1	3	4	2	3			1
*Streptococcus pneumoniae*			2	4				
*Streptococcus agalactiae*			3	1	1			
*Corynebacterium* *								
*Enterococcus faecium* *								
**Gram-negative bacteria**								
*Haemophilus influenza*	1	1	3	8			2	1
*Haemophilus parainfluenza* *								
*Proteus mirabilis*				1			1	
*Pseudomonas aeruginosa*	2			1	2		1	
*Moraxella catarrhalis*			3	2			1	
*Klebsiella pneumoniae*			1		1			
*Klebsiella oxytoca* *								
*Escherichia coli*			1	4	1			
*Serratia marcescens*				1				
*Citrobacter coseri* *								
*Enterobacter cloacae* *								
**Mycobacteria, n**								
*Mycobacterium tuberculosis* *								
*Nontuberculous mycobacteria* *								
**Fungi, n**								
*Aspergillus fumigatus* *								
**Virus, n**								
coronavirus								
adenovirus		1						
rhinovirus/enterovirus	1		1		1			
parainfluenza virus		2	2	1				

* not tested by multiplex PCR assay.

**Table 3:** (a) Comparison between traditional culture and multiplex PCR assay; (b) Comparison between traditional culture and multiplex PCR assay after exclusion of patients in whom only viruses were detected by PCR, and/or only bacteria by culture not included in the PCR panel.

(a)				
		**Biofire** ^®^		
		**positive**	**negative**	
**Culture**	**positive**	31	13	44
	**negative**	21	151	172
		53	163	216
(b)				
		**Biofire** ^®^		
		**positive**	**negative**	
**Culture**	**positive**	28	7	35
	**negative**	18	151	169
		46	158	204

**Table 4:** Comparison of BioFire^®^ FilmArray^®^ Pneumonia Panel to conventional culture for each microorganism, whereby in some patients multiple microorganisms were detected. P = positive, N = negative.

			Biofire^®^																			
			*Haemophilus*		*Staph aureus*		*Proteus*		*Pseudomonas*		*Strep. pneumoniae*		*Strep. agalactiae*		*Moraxella catarrhalis*		*Klebsiella pneumoniae*		*E. coli*		*Serrata marcescens*	
			P	N	P	N	P	N	P	N	P	N	P	N	P	N	P	N	P	N	P	N
**Culture**	***Haemophilus***	P	10	2																		
		N	6																			
	***Staph aureus***	P			7	1																
		N			6																	
	***Proteus***	P					1															
		N																				
	***Pseudomonas***	P							6	1												
		N																				
	***Strep. pneumoniae***	P									4	3										
		N									2											
	***Strep. agalactiae***	P																				
		N											4									
	***Moraxella catarrhalis***	P													2							
		N													2							
	***Klebsiella pneumoniae***	P																1				
		N															1					
	***E. coli***	P																	2	2		
		N																	4			
	***Serratia marcescens***	P																				
		N																			1	

**Table 5:** Multiple logistic regression with the dependent variable “positive culture” and the independent variables including sex, smoking (smoker versus never-smoker and ex-smoker) and CRP (<0.5 versus >0.5).

	OR	Lower CI	Upper CI	*p*-Value
Smoker	0.25	0.12	0.05	0.0001
CRP	2.29	1.13	4.63	0.0210
Sex	0.46	0.23	0.94	0.0331

## Data Availability

The data that support the findings of this study are available from the corresponding author, (D.G.), upon reasonable request.

## Supplementary Materials

The following supporting information can be downloaded at https://www.mdpi.com/article/10.3390/diagnostics15040469/s1: Table S1. Pathogens detected by PCR in 21 cases with negative traditional culture; Table S2. Pathogens detected by culture in 13 patients with negative PCR results.

## Abbreviations

COPD	Chronic obstructive pulmonary disease
COVID-19	Coronavirus disease
CRP	C reactive protein
ILD	Interstitial lung disease
PCR	Polymerase chain reaction
