# Supplementary material for: Determination and Comparison of the Pathogen Spectrum Evaluated by Microbial Culture and Multiplex PCR During Bronchoscopy with Regard to Clinical Utility of Routine Bronchial Wash in Patients with Various Pulmonary Diseases
Source: Diagnostics (Basel). 2025 Feb 14;15(4):469. doi: 10.3390/diagnostics15040469 (PMC11854491; doi:10.3390/diagnostics15040469)
Supplement: Supplementary file 1 [file diagnostics-15-00469-s001.zip › diagnostics-3429119-supplementary.pdf]

Supplemental material:

Table S1. Pathogens detected by PCR in 21 cases with negative traditional culture.

Patient	Pathogens
1	Haemophilus influenza, Staph. aureus
2	Rhinovirus/enterovirus
3	Haemophilus influenza
4	Stap. aureus
5	Stap. aureus, E. coli
6	Serratia marcescens
7	Rhinovirus/enterovirus
8	E. coli
9	Strep. agalactiae, Coronavirus
10	Haemophilus influenza, Strep. Pneumoniae
11	Staph. aureus
12	Moraxella catarrhalis, parainfluenca virus
13	Coronavirus
14	Klebsiella pneumoniae
15	Haemophilus influenza
16	Haemophilus influenza
17	Staph. aureus
18	Parainfluenca virus
19	Staph. aureus
20	Haemophilus influenza
21	Strep. agalactiae

Table S2. Pathogens detected by culture in 13 patients with negative PCR results.

Patient	Pathogens
1	Mycobacterium tuberculosis
2	Strep. pneumoniae
3	Haemophilus influenza
4	Strep. pneumoniae
5	Citrobacter coseri
6	Pseudomonas aeruginosa
7	Haemophilus influenza
8	Haemophilus parainfluenza
9	Aspergillus fumigatus
10	Enterobacter chloacae
11	Citrobacter coseri
12	E. coli
13	Klebsiella oxytoca
